# Regulation of Insulin Degrading Enzyme Activity by Obesity-Associated Factors and Pioglitazone in Liver of Diet-Induced Obese Mice

**DOI:** 10.1371/journal.pone.0095399

**Published:** 2014-04-16

**Authors:** Xiuqing Wei, Bilun Ke, Zhiyun Zhao, Xin Ye, Zhanguo Gao, Jianping Ye

**Affiliations:** 1 Department of Digestive Disease, Third Affiliated Hospital, Sun Yet-Sen University, Guangzhou, China; 2 Antioxidant and Gene Regulation Laboratory, Pennington Biomedical Research Center, Louisiana State University System, Baton Rouge, Louisiana, United States of America; Boston University School of Medicine, United States of America

## Abstract

Insulin degrading enzyme (IDE) is a potential drug target in the treatment of type 2 diabetes (T2D). IDE controls circulating insulin through a degradation-dependent clearance mechanism in multiple tissues. However, there is not sufficient information about IDE regulation in obesity. In this study, we test obesity-associated factors and pioglitazone in the regulation of IDE in diet-induced obese (DIO) C57BL/6 mice. The enzyme activity and protein level of IDE were increased in the liver of DIO mice. Pioglitazone (10 mg/kg/day) administration for 2 months significantly enhanced the enzyme activity (75%), protein (180%) and mRNA (100%) of IDE in DIO mice. The pioglitazone-induced changes were coupled with 50% reduction in fasting insulin and 20% reduction in fasting blood glucose. The mechanism of IDE regulation in liver was investigated in the mouse hepatoma cell line (Hepa 1c1c7 cells), in which pioglitazone (5 µM) increased IDE protein and mRNA in a time-dependent manner in an 8 h study. Free fatty acid (palmitate 300 µM) induced IDE protein, but reduced the mRNA. Glucagon induced, and TNF-α decreased IDE protein. Insulin did not exhibit any activity in the same condition. In summary, pioglitazone, FFA and glucagon directly increased, but TNF-α decreased the IDE activity in hepatocytes. The results suggest that IDE activity is regulated in liver by multiple factors in obesity and pioglitazone may induce IDE activity in the control of T2D.

## Introduction

Hyperinsulinemia is associated with obesity and is a risk factor for insulin resistance. Circulating insulin is determined by the balance of insulin clearance and secretion. A reduction in insulin clearance is a mechanism of hyperinsulinemia [1]–[3]. The role of insulin clearance is less investigated in the pathogenesis of hyperinsulinemia relative to insulin secretion in the obese models. There is controversy about the insulin clearance in obesity. In two recent studies, insulin clearance was enhanced in rodents by HFD in the first [4], but reduced in the second [5]. Insulin clearance was claimed as a target in the treatment of T2D given the role of hyperinsulinemia in the pathogenesis of insulin resistance [1]–[3]. Insulin resistance occurs after activation of the negative feedback loop of insulin receptor pathway in response to the high level insulin [1]. Serine kinases (Akt, PKC, mTOR, and S6K, etc) are involved in the feedback by inducing serine phosphorylation of insulin receptor substrates (IRSs) [1], [6]–[8]. Induction of IDE activity is expected to prevent the negative feedback by lowering circulating insulin. However, there is not sufficient information about IDE regulation in obesity [9], [10]. In this study, we investigated IDE activity in response to obesity-associated factors and pioglitazone.

IDE is a rate-limiting enzyme in the insulin degradation process [11]. It is an intracellular 110–kDa thiol zinc-metalloendopeptidase located in the cytosol, peroxisomes, endosomes, and cell surface. IDE catalyzes degradation of several small proteins including insulin, amylin, β-amyloid protein, etc. [12]. IDE enzyme activity is induced by zinc, inhibited by copper, aluminum [13], and nitric oxide [14]. Inactivation of IDE by gene knockout induces hyperinsulinemia and insulin resistance in mice [15]. Pioglitazone is an insulin sensitizing medicine that reduces blood insulin and improves systemic insulin sensitivity. It is generally believed that the control of hyperinsulinemia is a result of improved insulin sensitivity in the peripheral tissues [16], [17]. Pioglitazone leads to insulin sensitization in liver and skeletal muscle by reducing circulating lipids through activation of PPARγ in the fat tissues [18], which stimulates adipocyte differentiation and small adipocyte generation in the subcutaneous fat pads. Although pioglitazone reduce circulating insulin, it is not clear if IDE is involved. To address this question, we investigated IDE (insulin) in the liver of DIO mice following pioglitazone treatment.

In this study, we observed that IDE enzyme activity and protein were induced in liver by HFD in DIO mice. In the obesity-associated factors, FFA and glucagon elevated, but TNF-α decreased IDE protein. The IDE activity was enhanced by pioglitazone in DIO mice, suggesting a new mechanism of thiazolidinedione action in the control of insulin resistance.

## Methods

### Reagents

Rabbit polyclonal antibody to insulin degrading enzyme (IDE, Cat. ab32216) and mouse monoclonal to β-Actin (Cat. ab6276) were from Abcam (1 Kendall Square, Suite B2304 Cambridge, MA 02139–1517, USA). Secondary antibodies include ECL Anti-rabbit IgG horseradish peroxidase linked whole antibody (from donkey, Cat. NA934V, and ECL Anti-mouse IgG horseradish peroxidase linked whole antibody (from sheep, Cat. NA931V, GE Healthcare UK limited, Little Chalfont Buckinghamshire, HP7 9NA UK). Immobilon-P^SQ^ PVDF Transfer Membranes (Cat. ISEQ 10100) and Chemiluminescence reagent Luminata Western HRP Substrate (Cat. WBLUF0100) were from Millipore (Billerica, MA 01821). The X-ray film was from Phenix Research (Cat. F-BX810, Candler, NC, USA).

### Diet-induced Obese (DIO) Model

All animal experiments were approved by the Institutional Animal Care and Use Committee at the Pennington Biomedical Research Center. DIO model was generated as described elsewhere [19]. C57BL/6 mice of 6–8 weeks were purchased from Jax Lab and fed a high fat diet (HFD; 58% kcal in fat, D12331; Research Diets, New Brunswick, NJ) for six months to induce type 2 diabetes. Regular Chow diet was used in the control group. Pioglitazone (Actos, 10 mg/kg/day) was administrated through dietary supplement for 2 months after 4 month HFD-feeding. Mice were sacrificed after 4 hour fasting. The liver samples were collected and maintained at −80°C degrees. The serums were collected and maintained at −20°C degrees.

### Cellular Model

The mouse hepatoma cell line (1c1c7) was obtained from American Type Cell Culture and maintained in our lab. Cells were cultured and maintained in Dulbecco’s Modified Eagle’s Medium (DMEM) with 10% fetal bovine serum (Cat. S11150, Atlanta Biologicals, Inc. Lawrenceville, GA, USA) and maintained at 37°C with 5% CO_2_/air atmosphere. All the cells were passaged once per two days and routinely examined for mycoplasma contamination. When the cell reached a 90% confluence in the 10 cm plate, it was used in experiments.

5×10^5^ cells were loaded to each well of the six-well plates 12 hours before experiments; then starved overnight in Dulbecco’s Modified Eagle’s Medium (DMEM) containing 0.25% BSA. The cells were treated with 300 µM BSA-conjugated palmitic acid (Cat. P9767, Sigma) for 1, 2, 4, and 8 hours. BSA was used in the control. The cells were treated with 200 nM insulin (Cat. I9278, Sigma) or 5 µM pioglitazone (Cat. E6910, Sigma). DMSO was used 1∶1000 in the control for pioglitazone.

### IDE Activity Assay

Liver tissue extracts were prepared by homogenizing tissue in Cytobuster Protein Extraction Reagent (Cat. 71009-3, EMD Millipore, Billerica, MA 01821) according to the manufacturer’s recommended protocol. IDE activity was assessed with the InnoZyme Insulysin/IDE Immunocapture Activity Assay Kit (Cat. CBA079, Calbiochem/Millipore) and was normalized to the value of control group. Relative IDE activity was expressed in this study.

### Western Blotting

The protein preparation and the immunoblotting were described elsewhere [19], [20]. Briefly, the whole-cell lysate was made in a lysis buffer under sonication, which breaks both cytoplasmic and nuclear membranes. All of the immunoblotting experiments were conducted at least three times. To make a compare, the intensity of the protein signal was analyzed quantitatively using Image J software (National Institutes of Health, Bethesda, MD). The ratio of IDE signal over the β actin signal was used to express the quantification result.

### Quantitative Real-time RT-PCR (qRT-PCR)

TaqMan RT-PCR reaction was used to quantify mRNA of IDE. The total RNA was prepared from cell lysates or tissues with Trizol reagent (Cat. T9424, Sigma, St. Louis, MO) as described elsewhere [19], [20]. The assay was conducted using the 7900 HT Fast real-time PCR System (Applied Biosystems, Foster City, CA). The target mRNA signal was normalized with ribosome 18S RNA. IDE (Mm00473077) primer and probe were from the Applied Biosystems.

### Plasma Glucose and Insulin

The mice were fasted over night with free access to water. Blood glucose was monitored in the tail vein blood using the FreeStyle blood glucose monitoring system (TheraSense, Phoenix, AZ). Insulin was determined using a Mouse Serum Adipokine multiplex Kit (Cat. MADPK-71K, Millipore) according to the manufacturer’s instruction.

### Statistical Analysis

Student**-** *t* test was performed in data analysis with significance at *P*<0.05.

## Results

### Induction of Hepatic IDE by HFD

**Figure 1:**
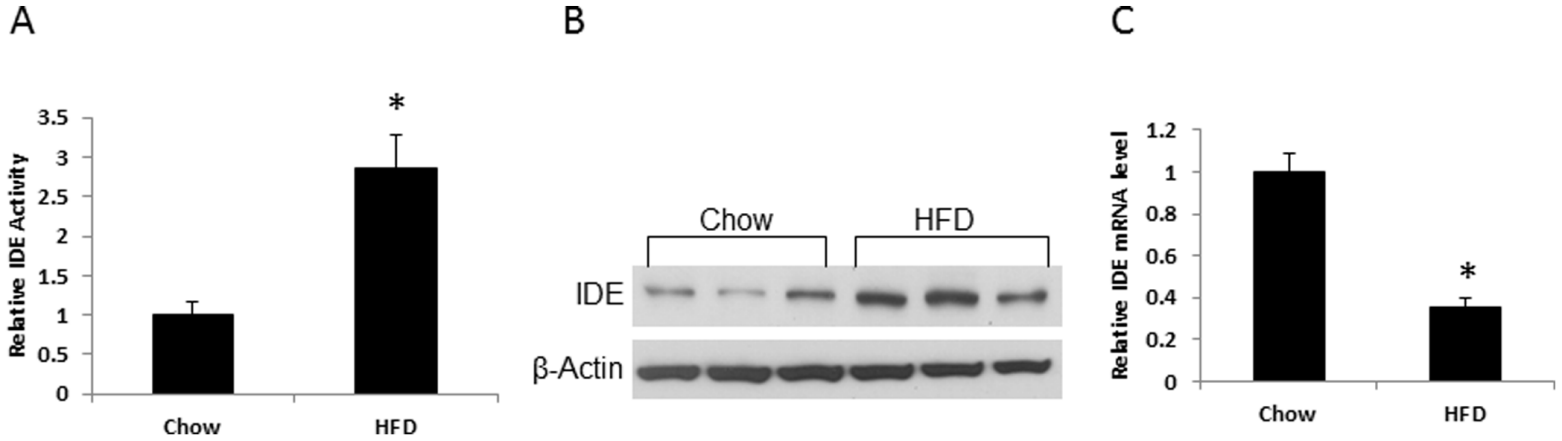
IED in liver tissue of DIO mice. The mice were on chow diet or high fat diet (HFD) for 6 months, and the samples were collected in 4 h fasting state. A. Enzyme activity of IDE. HFD increased enzyme activity of IDE that was determined via the InnoZymeTM Insulysin/IDE Immunocapture Activity Assay Kit (n = 10). B. IDE protein. HFD increased IDE protein level in Western blot. The experiment was performed three times with consistent results, and a representative blot is shown. C. IDE mRNA in liver (n = 4). *P<0.05 compared with the control.

We investigated insulin clearance in DIO model by examining hepatic IDE activity. Liver is a primary organ in insulin clearance and it is responsible for removal of 70% secreted insulin from islet. A reduction in the insulin clearance function contributes to hyperinsulinemia. IDE is a major rate-limiting enzyme for insulin clearance. Inactivation of IDE activity leads to hyperinsulinemia in gene knockout mice [15]. Hyperinsulinemia is a character of DIO mice, and the role of IDE in the hyperinsulinemia remains largely unknown. To address this question, we examined IDE activity in enzyme function, protein and mRNA in the liver tissue of DIO mice. The enzyme activity was determined in the liver tissue using an enzyme assay kit. The activity was increased in the DIO mice by 186% (Fig. 1A). This change was associated with a 150% increase in IDE protein in a Western blot (Fig. 1B). However, mRNA of IDE was reduced by 65% in qRT-PCR test. The data suggest that IDE activity was enhanced by the high fat diet in the liver of DIO mice.

### Induction of Hepatic IDE by Pioglitazone

**Figure 2:**
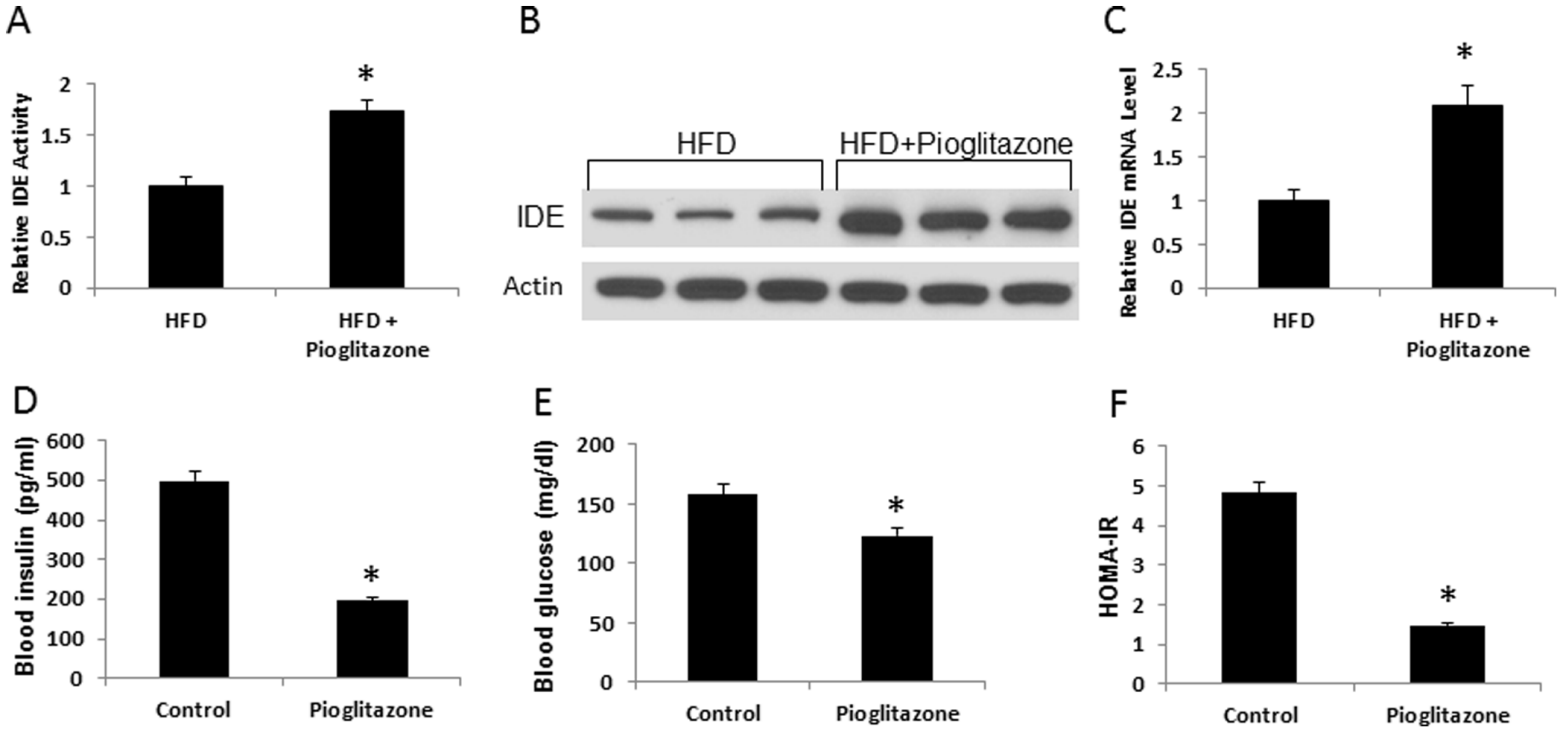
Regulation of liver IDE by pioglitazone. A. IDE enzyme activity. Liver tissue was collected from DIO C57BL/6J mice were treated with pioglitazone (10 mg/kg/d) for last 2 months in 6 month HFD feeding. The samples were collected in fasting state. The IDE enzyme activity was determined (n = 10). B. IDE protein. The protein level was determined in Western blot. The experiment was performed three times with consistent results, and a representative blot is shown. C. IDE mRNA. mRNA level was determined in qRT-PCR (n = 5). D. Fasting blood insulin. E. Fasting blood glucose. F. HOMA-IR index (n = 10). *P<0.05 compared with the control.

Pioglitazone is a thiazolidinedione (TZD)-derived insulin-sensitizing medicine. It is generally believed that Pioglitazone reduces hyperinsulinemia through improvement of peripheral insulin sensitivity. However, one study suggests that rosiglitazone (a similar insulin-sensitizing medicine derived from TZD) may enhance insulin clearance in patients [21]. Unfortunately, IDE activity was not examined in the study. To address this issue, we examined IDE activity in the liver tissues of DIO mice after 2 month pioglitazone treatment at a dosage of 10 mg/kg/day. The enzyme activity of IDE was increased by 75% in the pioglitazone-treated mice (Fig. 2A). The IDE protein was enhanced by 190% (Fig. 2B) and mRNA was increased by 100% (Fig. 2C). Pioglitazone reduced fasting insulin, fasting glucose and HOMA-IR index in DIO mice (Fig. 2, D–F), suggesting an improvement in insulin sensitivity. The data suggests that pioglitazone administration enhances IDE activity in the liver during improvement of insulin sensitivity.

### Pioglitazone Induction of IDE Expression in Hepatic Cell Line

**Figure 3:**
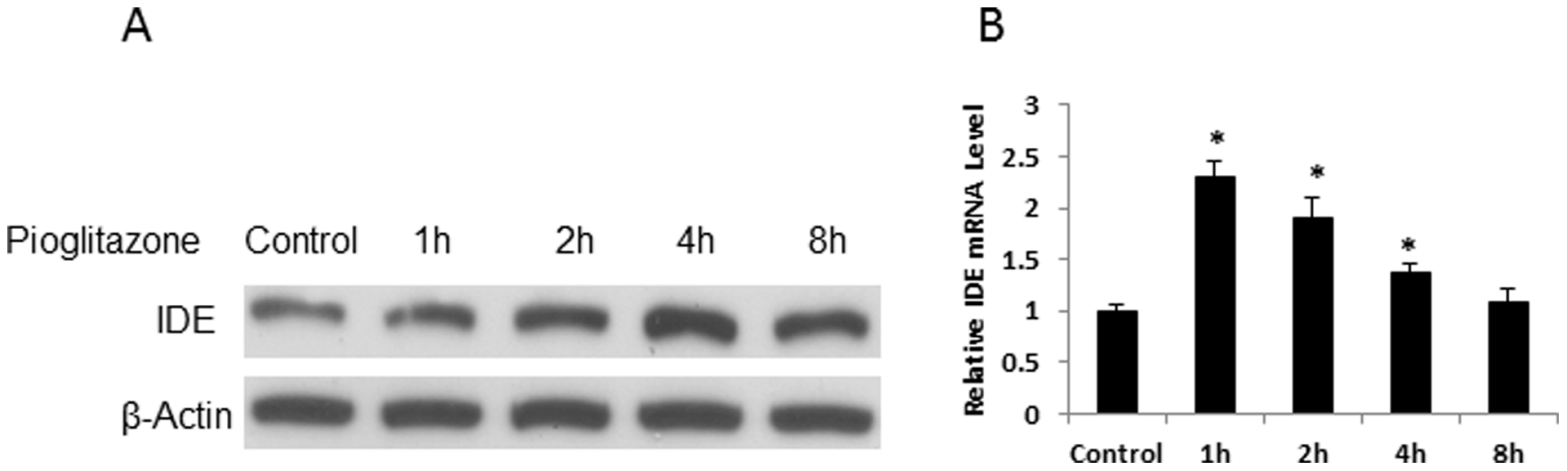
Regulation of IDE in hepatocytes by pioglitazone. A. IDE protein. Hepatocytes (1c1c7) were treated with pioglitazone (5 µM) for 8 hours. In the control, vehicle (DMSO) was added at 1∶1000 into the culture medium for 8 hour treatment. The experiment was performed three times with consistent results, and a representative blot is shown. B. IDE mRNA. mRNA was determined by qRT-PCR (n = 3), *P<0.05 compared with the control.

Cellular studies were conducted to understand the mechanism of IDE regulation in the liver. We examined IDE activity in the mouse hepatoma cell line 1c1c7 in cell culture after pioglitazone treatment. 1c1c7 is widely used as a model of hepatocytes. In this study, the cells were treated with 5 µM pioglitazone for 8 hours. IED protein and mRNA were determined at multiple time points in the time-course study. IDE protein was increased by pioglitazone in a time-dependent manner in the first few hours with a peak at 4 hours (Fig. 3A). The increase disappeared thereafter at 8 hours. IDE mRNA exhibited an increase, but in a different time course. The mRNA was peaked at 1 hour and gradually reduced thereafter (Fig. 3B). This group of data suggests that pioglitazone directly induced IDE expression in hepatocytes in a time-dependent manner. The induction was observed in both IDE mRNA and protein.

### Induction of IDE Protein by Free Fatty Acid (FFA)

**Figure 4:**
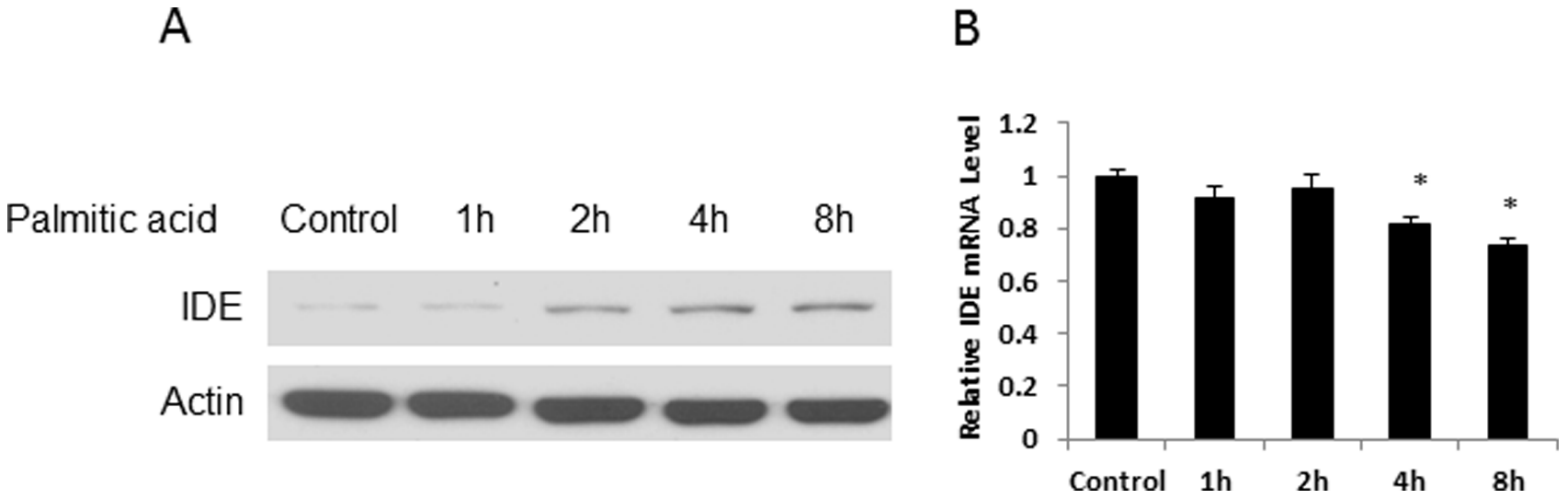
Regulation of IDE by free fatty acid. A. IDE protein. 1c1c7 cells were treated by palmitic acid (300 µM) for different times. In the control, cells were treated with BSA at the equal concentration for 8 hours. IDE protein was determined in whole cell lysate in Western blot. The experiment was performed three times with consistent results, and a representative blot is shown. B. IDE mRNA. IDE mRNA was in qRT-PCR (n = 3). *P<0.05 compared with the control.

IDE protein was enhanced in mouse liver by HFD in this study (Fig. 1). The mechanism was not known. We addressed this issue by testing IDE in 1c1c cells following the palmitic acid treatment as long chain fatty acids are the main component in HFD. IDE protein was increased in a time-dependent manner by the palmitate acid (300 µM) with the highest level at 8 hours (Fig. 4A). However, IDE mRNA was not increased in the same condition. Instead, mRNA was decreased at 4 and 8 hours in the presence of FFA (Fig. 4B). This pattern of changes resembles those observed in the liver of DIO mice. The data suggest that FFA may directly up-regulate IDE protein in hepatocytes.

### Insulin did not Regulate IED in Hepatocytes

**Figure 5:**
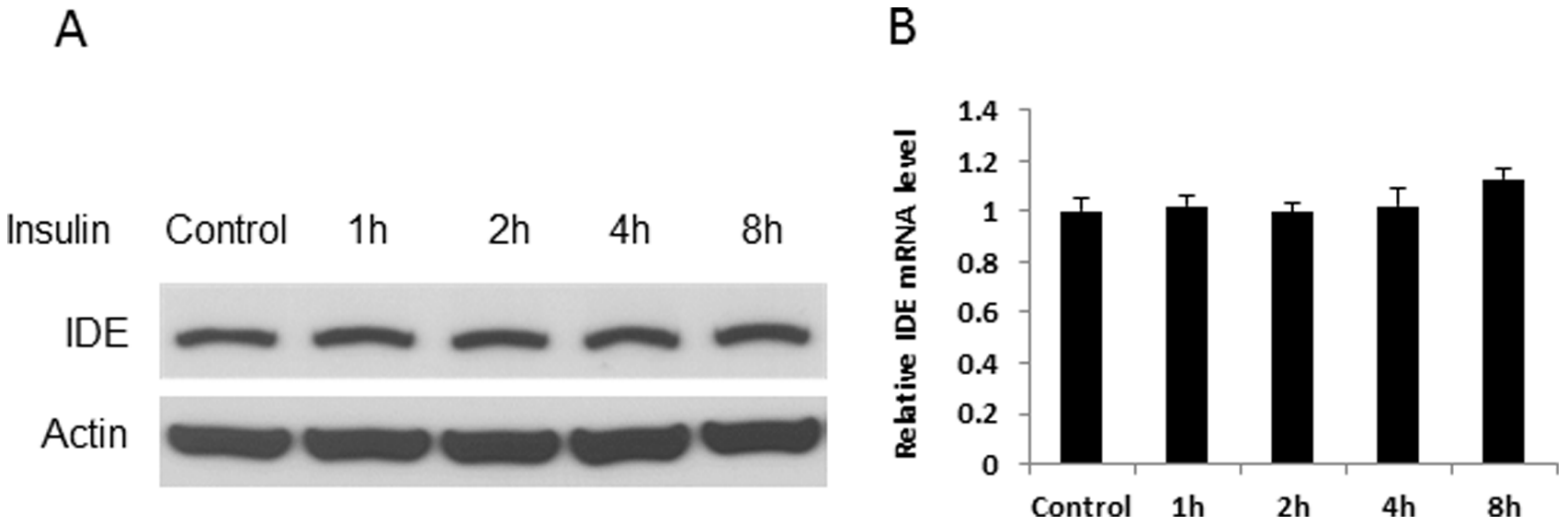
Regulation of IDE by insulin. A. IDE protein. 1c1c7 cells were treated by insulin (200 nM) for 8 hours. IDE protein was determined in the whole cell lysate in Western blot. In the control, PBS was added into the culture medium in the 8 hour treatment. The experiment was performed three times with consistent results, and a representative blot is shown. B. IDE mRNA. mRNA was determined by qRT-PCR (n = 3).

Regulation of IDE activity by insulin is of interesting as there is hyperinsulinemia in obesity. However, the relationship remains to be established. To address this question, we examined IDE activity in 1c1c7 cells after the insulin treatment. The cells were treated with 200 nM insulin for 8 hours. IDE activity was determined in protein and mRNA. No significant change was observed in IDE in the insulin-treated cells (Fig. 5, A and B), suggesting that insulin may not directly regulate IDE expression in hepatocytes.

### Induction of IDE Protein by Glucagon and Forskolin

**Figure 6:**
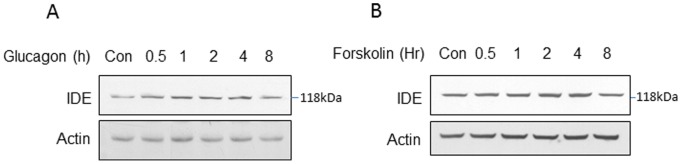
Regulation of IDE by glucagon. A. IDE protein induction by glucagon. 1c1c7 cells were treated with glucagon (100 ng/ml) for 8 hours. IDE protein was determined in the whole cell lysate at different times in Western blot. In the control, PBS was added into the culture medium in the 8 hour treatment. B. IDE protein induction by forskolin (10 µM). IED protein was determined in the whole cell lysate at different times in Western blot. The experiment was performed three times with consistent results, and a representative blot is shown.

An increase in glucagon activity contributes to hyperglycemia in obesity [22]. There is no literature about IDE regulation by glucagon according to our knowledge. The glucagon activity was tested in 1c1c cells. IDE protein was induced by glucagon in a time-dependent manner in an 8 hour study. IDE protein was induced by 70% at 30 mins and a peak of increase at 100% was observed at 1 hour (Fig. 6A). The elevation was maintained for 4 hours and then decreased at 8 hours. A similar pattern of IDE protein increase was observed in the cells treated with forskolin, an activator of protein kinase A (PKA), suggesting that glucagon induced IDE protein through activation of the cAMP/PKA signaling pathway.

### Inhibition of IDE Protein by TNF-α

**Figure 7:**
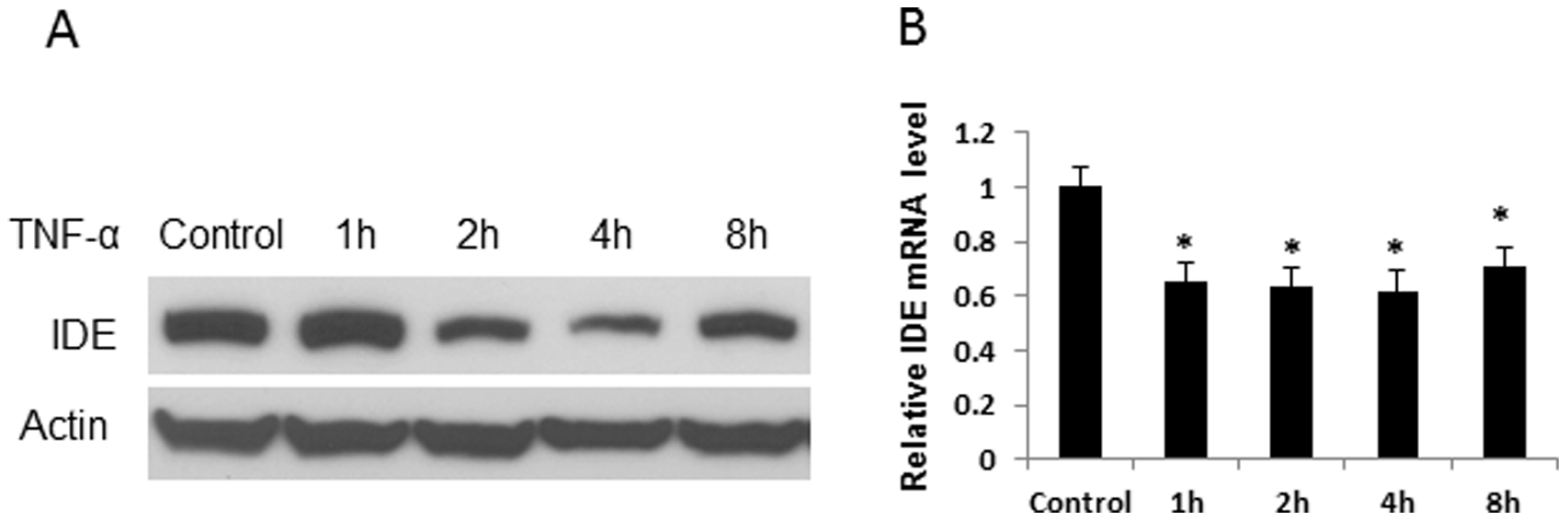
Regulation of IDE by TNF-α. A. IDE protein. 1c1c7 cells were treated with TNF-α (10 ng/ml) for 8 hours. Equal volume of PBS was added into the culture medium in the control for 8 hours. IDE protein was determined at different times as shown and the experiment was performed three times with consistent results. A representative blot is shown. B. IDE mRNA (n = 3). *P<0.05 compared with the control group.

Chronic inflammation is associated with obesity and liver is one of the inflammatory sites next to the white adipose tissue [23]. There is no study about regulation of IDE by inflammation in obesity models. We addressed this issue by testing IDE in hepatocytes following treatment with TNF-α, a representative pro-inflammatory cytokine. IDE protein was modestly increased by TNF-α at 1 hour and then decreased significantly thereafter in the 8 hour treatment (Fig. 7A). IDE mRNA was consistently reduced by TNF-α in hepatocytes. The data suggests that the major activity of TNF-α is to inhibit IDE expression in hepatocytes.

## Discussion

Our study suggests that pioglitazone has a potential activity in the induction of IDE activity. IDE is a potential drug target in the treatment of type 2 diabetes. Up-regulation of IDE activity may help to control hyperinsulinemia in the disease. The liver removes 50% insulin peptide in the portal vein during the first passage [9]. Insulin removal is dependent on receptor-mediated uptake and IDE-catalyzed degradation of insulin in several tissues, in which liver is a major one [9]. Insulin clearance is impaired in obesity [24] and a defect in IDE activity is proposed as a major factor [9]. Knockout of IDE gene leads to hyperinsulinemia in mice from impaired insulin clearance in multiple tissues [15], [25]. IDE gene mutation is responsible for hyperinsulinemia and insulin resistance in the Goto–Kakizaki rats (a genetic model of non-insulin-dependent diabetes) [26]. Human studies suggest that polymorphisms of IDE gene is closely associated with a high risk for type 2 diabetes [27], [28]. IDE malfunction contributes to insulin resistance and hyperglycemia in many cases although not all [9]. Up-regulation of IDE activity is an ideal approach in the treatment of insulin-mediated insulin resistance in T2D. Unfortunately, not much is known about IDE regulation in obesity [9].

We observed that pioglitazone induced IDE enzyme activity in the liver of DIO mice, which was associated with an increase in IDE protein and mRNA. Although IDE catalyzes degradation of insulin and β-amyloid peptide, current knowledge about IDE regulation is mostly derived from the study of β-amyloid peptide in the Alzheimer disease field. IDE takes care of β-amyloid clearance and IDE dysfunction is a critical factor in the pathogenesis of Alzheimer disease. In the current study, we observed that pioglitazone induced IDE protein and mRNA in vivo and in vitro. Consistently, the IDE gene promoter contains a PPARγ binding site, and PPARγ induces the gene transcription in neuronal cells [29]. Regulation of IDE expression represents a novel activity of pioglitazone in the pharmacological action. This activity suggests that pioglitazone may reduce hyperinsulinemia through accelerating clearance of insulin. Our study supports the role of PPARγ in the regulation of IDE expression. IDE gene transcription was reported to be induced by NRF1 [30] and reduced by Notch signaling proteins HES-1 or Hey-1 in neuronal cells [31]. In addition, pioglitazone may increase IDE through inhibition of TNF-α expression.

Our study suggests that FFA (palmitate) and glucagon induces IDE protein. We examined FFA, insulin and glucagon to understand IDE change in the liver of DIO model. Our data suggests that FFA and glucagon induce IDE protein. Early reports suggest that FFA reduces IDE activity and insulin clearance in cell cultures [32], [33]. The FFA effect remains to be verified with well-designed experiments in vivo. Two human studies report opposite effects of high fat diet on insulin clearance. Cafeteria diet was reported to enhance the insulin clearance in the first study [4], but reduced in the second study [5], suggesting that the FFA activity remains unclear in vivo. In this study, we observed that IDE protein and enzyme activity were induced in the liver of DIO mice. However, IDE mRNA was reduced. FFA exhibited the same effect in cell culture, suggesting that FFA directly induces IDE protein in hepatocytes. The molecular mechanism of FFA activity remains to be identified. Gene expression may not involve as FFA did not induce IDE mRNA. Protein modification may play a role, and IDE protein is regulated by ubiquitin [34]. The FFA signaling pathway remains to be identified in the IDE regulation. FFA activates protein kinase C (PKC), JUN c-terminal kinase (JNK), and IkBα kinase (IKK) pathways [35]. The role of PKC, JNK and IKK pathways are not supported in IDE up-regulation due to the TNF-α inhibition of IDE protein and mRNA in this study. Glucagon induced IDE protein in this study. Activation of cAMP/PKA pathway by forskolin exhibited the same effect as glucagon. Therefore, the cAMP pathway is likely involved in IDE up-regulation by glucagon. The physiological significance is that IDE likely involve in a feedback regulation of glucagon. It was reported that IDE protein was induced by 25% by insulin in the primary hippocampal neurons [36]. However, such an effect was not observed in the liver cell line in the current study. Insulin was reported to inhibit IDE enzyme activity in an early study [37], but induce the activity in a later study [38]. We cannot exclude insulin activity in the regulation of IDE enzyme activity as it was not examined in this study.

Our data suggests that TNF-α inhibits IDE activity in 1c1c cells. The mechanism is not known. We propose that activation of NF-kB by TNF-α may play a role. NF-kB is known to suppress PPARγ function through multiple mechanisms [17], [39]. Pioglitazone may antagonize the TNF-α activity by activation of PPARγ. In addition, inhibition of TNF-α expression by pioglitazone may be another mechanism of pioglitazone action. Thiazolidinediones including pioglitazone have anti-inflammatory effects in macrophages [40], [41].

This is a preliminary study and the observations need to be confirmed with more sophisticated assays or model systems in the future. The assay used to measure IDE enzyme activity is a FRET-based assay using a 9 amino acid peptide. The activity remains to be tested using full length insulin as a substrate as IDE activity toward insulin and small peptides is not the same [42]. We show that IDE levels and activity are altered by a number of stimuli. The results are based on mRNA and protein levels of IDE in cellular models, and the IDE enzyme activity remains to be tested in insulin degradation. In addition, different fatty acids such as mono-unsaturated fatty acid (oleic acid) and poly-unsaturated fatty acids (DHA and EPA) remain to be tested in the regulation of IDE.

In summary, we examined several factors in the regulation of IDE activity. In the multiple-step process of insulin clearance, IDE defect may impair insulin clearance and increase the risk for hyperinsulinemia. Fatty liver and chronic inflammation may contribute to the pathogenesis of T2D through impairing IDE activity. The IDE activity was induced by pioglitazone, suggesting a new action of the medicine in the treatment of T2D. Although the study was conducted in liver and hepatocytes, the observation may apply to IDE regulation in other tissues such as kidney, brain and fat tissues.
